# Map Learning in Aging Individuals: The Role of Cognitive Functioning and Visuospatial Factors

**DOI:** 10.3390/brainsci11081033

**Published:** 2021-08-03

**Authors:** Veronica Muffato, Laura Miola, Francesca Pazzaglia, Chiara Meneghetti

**Affiliations:** 1 Department of General Psychology, University of Padova, 35131 Padova, Italy; laura.miola@phd.unipd.it (L.M.); francesca.pazzaglia@unipd.it (F.P.); chiara.meneghetti@unipd.it (C.M.); 2 Interuniversity Research Center in Environmental Psychology (CIRPA), 00185 Rome, Italy

**Keywords:** map learning, cognitive functioning, MoCA, older adults, spatial anxiety, sense of direction

## Abstract

Aging coincides with a decline in map learning ability, but it is unclear to what extent different aspects of the mental representation are susceptible. The present study aimed to investigate knowledge about landmarks, their positions and distances (categorical and distance relations, respectively) in relation to aging as well as cognitive functioning (measured with the Montreal Cognitive Assessment [MoCA]), visuospatial abilities, and self-reported wayfinding inclinations. Thirty young adults and 60 older adults (30 aged 63–74 and 30 aged 75–86) learned a map, freely recalled the landmarks and performed a map drawing task (considering the number of landmarks missing, position accuracy and distance accuracy). Before that, older participants were also assessed regarding their general cognitive functioning (MoCA) and a series of visuospatial measures. The results show age-related differences among adults in recalling landmarks and in both categorical and distance relations, with a worsening of performance of old-olds only in the former. Older adults’ MoCA score related to accuracy in the three measures, and an additional role of spatial anxiety was found for distance accuracy. Above and beyond the age-related decline, the quality of older people’s spatial mental representation is related to higher general cognitive level and lower spatial anxiety.

## 1. Introduction

The ability to acquire knowledge about environments is an important everyday activity. One of the modalities to acquire such knowledge is from a map. Take the case in which you must reach a point of interest in an unfamiliar city; reading a map is a common way to understand which roads to take. Maps depict the environment from an aerial point of view, showing the position of landmarks in relation to one another, and with a defined distance between them [1]. Maps are figural space sources (Montello, 1993), given that a map symbolically reproduces a large area, such as a city, on a smaller scale with respect to an individual’s body and can be perceived all at once, as a whole, from one point of view.

Consequently, studying populations that show difficulties in learning a new environment from a map is of particular interest. One such population is older adults. Map learning issues in older adults have been amply shown when comparing them with younger adults (e.g., [2,3,4,5]). However, the use of maps in aging individuals seems less susceptible to age-related effects than other types of input to learn the environment, such as navigation [6,7] or spatial descriptions [8]. In addition, the use of maps in aging individuals seems less susceptible to age-related effects when a task resembling the same format of the learning phase is used, as in the case of drawing a map of the environment [4]. Muffato et al. [4], using a sketch map drawing task after map learning in young and older adults, showed that older adults had difficulty remembering the location of some of the landmarks (i.e., older adults inserted fewer landmarks into the map compared with younger people), but they arranged the ones they could recall in their appropriate relative positions in the layout as a whole. Thus, only some aspects of mental representations seem to deteriorate in aging after learning a map, such as the number of missing landmarks, whereas other aspects could be less susceptible to decline, such as the position of landmarks relative to one another (above/below, left/right). This information about inter-landmark positions is defined as categorical spatial relations. Such categorical relations and distance relations (the inter-object distances, near to/far from) are two aspects related to the quality and precision of a spatial mental representation [9]. Previous research has shown a decrease in performance regarding distance relations with aging and lower age-related differences in categorical spatial relations [10], even when testing mental representations of environments familiar to participants [11]. However, these two aspects of the mental representation quality—position accuracy (categorical relations) and distance accuracy (distance relations)—have not yet been analyzed after map learning in aging individuals. Map learning as a source differs from other types of spatial leaning inputs, such as learning from navigation, which has a scale larger than the body and that requires the integration of information over time and from a person’s point of view (environmental space [2]). Both types of input (map leaning and navigation) prompt spatial mental representations of the environment [12], but their features may differ. Thus, it is worth focusing on the analysis of map learning, especially in aging individuals given older adults’ greater difficulty with forming mental representations and representing information allocentrically [13]. A deep investigation of a person’s mental representation characteristics after he or she learns a map can help with understanding whether aging is linked to how many landmarks older adults can recall (recalling information about landmark identity), how many of them they feel confident to position landmarks on a sketch map (recalling information about landmark positioning), the accuracy of their positioning relative to the other landmarks (categorical relations), and the precision of the inter-landmark distance (distance relations). When accurate, this information is a prerequisite for successful navigation [14]; thus, this issue merits deep investigation in aging individuals.

Other than age, several individual factors can play a role in the quality of mental representation, such as visuospatial factors [12,15]. They include both abilities (objective measures) and preferences and attitudes (subjective inclinations) related to the performance in the environment. Among the visuospatial abilities is visuospatial working memory (VSWM), which retains and processes visuospatial information [16], as well as mental rotation and perspective-taking abilities, the higher-order abilities to mentally rotate objects and to adopt different views, respectively [17,18]. Among the preferences and attitudes is an individual’s self-rated sense of direction and spatial anxiety, defined as finding spatial demands worrying [19]. The objective measures are liable to decline with increasing age, whereas the latter are not susceptible to change over time (e.g., [20]). Such abilities have been found to be important for good navigation performance [21], and even for map learning ability in young and older adults [4]. Another factor related to the individual that can play a role in the quality of older adults’ mental representation is their general level of cognition. A common measure used in aging studies as a measure of one’s cognitive level is the Montreal Cognitive Assessment (MoCA) [22]. It assesses several cognitive domains, such as short-term and delayed memory recall, visuospatial abilities, executive functioning, attention and concentration, language, and orientation to time and place [22,23], but a cutoff score on the total score is usually used for cognitive screening purposes. Although it is common to find it used as an inclusion criterion in spatial domain studies in aging, a paucity of studies has related it with spatial performance [24,25]. These studies found—after learning an environment from a video navigation—a superiority of people with a higher general cognitive level in tasks such as judging the directions of landmarks and locating landmarks on a map, whereas no differences emerged in recalling the sequence of landmarks and in a route repetition task (using the same first-person view as in the learning condition). These studies provided a first indication that general cognitive level—as measured with the MoCA score—might relate to spatial performance, depending on the type of spatial request. It is not clear, however, if the role of the cognitive level emerges when one asks older adults to solve tasks that require a change in perspective, such as from the first-person—egocentric—perspective of navigation learning to map-based—allocentric—knowledge when judging landmark directions and positions [24,25,26], or if it emerges when tasks require map-based (allocentric) knowledge per se (judging landmark directions and positions as in a map drawing task) independently from the egocentric/allocentric point of view of the learning phase (navigation/map learning). So far, no study has investigated the role of the cognitive level as measured with the MoCA scores in map learning in aging individuals, which could disentangle this issue.

The present study was thus aimed at investigating the quality of mental representation using the free recall of landmarks and a map drawing task [27] after map learning takes place in aging individuals. Map learning is important in aging individuals given that—although it is a tool that makes spatial information directly accessible by showing landmarks and their relationships from an aerial point of view—it is an ability that is susceptible to decline. The goal was therefore to investigate information about landmarks (in terms of both the number of landmarks recalled and the number of landmarks inserted into the map drawing task), their positions (categorical spatial relations), and their distances (distance relations [28]) in young, young-old, and old-old people. Individual factors were also investigated for their potential roles in the quality and precision of mental representation in aging individuals. Among these factors, the roles of the cognitive level as measured with the MoCA score, VSWM, and visuospatial abilities (rotation and perspective-taking abilities) were considered as objective measures. In addition, self-reported wayfinding attitudes (sense of direction and spatial anxiety) were considered as subjective measures. The present study is novel in that it focused on investigating all of these factors (the cognitive level as measured with the MoCA, VSWM, rotation abilities, and self-reported visuospatial attitudes) together–and after map learning. This will shed more light on the issue of age-related changes in mental representations after one learns a map.

Concerning information about landmarks, we hypothesized that the general memory impairment that comes with aging [29] could cause difficulty with the recall of the landmarks learned from a map in older adults compared with young people, with a worsening taking place between young-olds and old-olds [30]. Concerning the categorical and distance relations, age-related effects are expected, with a more pronounced worsening with increasing age for distance relations, as found with other learning inputs (e.g., [11]). However, here, for the first time, categorical and distance relations were analyzed after map learning. Given that a map provides direct access to landmarks’ relationships to one another [1], age-related differences could be attenuated or not present. This is also assumed given previous studies reporting inconsistent results for map drawing performance after the learning of spatial information from a map in aging. Some studies reported similar levels of performance in young and older adults [7,8], whereas others found impaired performance in older adults [5,6]. Concerning older adults’ individual factors, we supposed that the general cognitive level as measured with the MoCA score [25,27], the visuospatial abilities, and the visuospatial wayfinding inclinations [4] could relate to spatial mental representation characteristics. The role of the subcomponents of the MoCA scores are also explored here in terms of their relationship with mental representation accuracy.

## 2. Materials and Methods

### 2.1. Participants

The study involved 60 healthy and independently living old adults, 30 of whom were young-old, from 63 to 74 years (15 females; *M* age = 68.73, *SD* = 3.33), and 30 older adults aged from 75 to 86 years (15 females; *M* age = 78.50, *SD* = 3.17). The study also involved 30 young adults (15 females; *M* age = 23.53, *SD* = 2.34) as a control of age-related differences in the various measures. Based on power analyses run with the “pwr” library in R for linear models, we calculated that 51 older participants were needed to obtain a power of 0.80 and an effect size of 0.30. All participants were volunteers recruited by word of mouth at recreation centers (for older adults). For older adults, an inclusion criterion of a MoCA [21] score of at least 22 was set to include typically aging individuals (see [23] for the Italian normative sample). Participants had all attended the compulsory level of schooling (young: *M* age of education = 13.77, *SD* = 1.25; young-old: *M* age of education = 10.73, *SD* = 2.34; older adults: *M* = 10.57, *SD* = 3.46), with young adults having undergone more years of education—*F*(2,87) = 15.33, η^2^_p_ = 0.26, *p* < 0.01—compared with the older adult groups, which did not differ between each other (*p* = 0.80). None of the participants had a history of psychiatric, neurological, or other diseases capable of causing cognitive, visual, auditory, or motor impairments [31].

The local ethics committee approved the study, and all participants were informed about its purposes and gave their written informed consent in accordance with the Declaration of Helsinki [32].

### 2.2. Materials

#### 2.2.1. Individual Cognitive and Self-Reported Measures

Montreal Cognitive Assessment (MoCA, [22]; showing a good reliability of 0.83). The MoCA assesses multiple aspects, such as executive functioning (short trail-making B, phonemic fluency task, a two-item verbal abstraction task), visuospatial abilities (clock-drawing task, cube copy), attention and working memory (sustained attention task, a serial subtraction task, and a digit forward and backward task), language (naming task, repetition of two syntactically complex sentences), short-term and delayed memory recall (after 5 min), and orientation in time and place (max score 30).

Jigsaw Puzzle Test (JPT, [33]; good reliability of *r* = 0.83). The JPT involves mentally recomposing puzzles of objects divided into different levels of difficulty (from 2 to 10 pieces). Participants must solve at least two of the three puzzles at a given difficulty level in order to proceed to the next. The final score is the sum of the difficulty levels of the last three puzzles solved (max 29).

Short Mental Rotations Test (sMRT, [34], Cronbach’s alpha = 0.81; adapted from [15]). The sMRT consists of finding two of four objects (3D assembled cubes) that match a target object in a rotated position (10 items; 5-min time limit; score 0–10).

Short Object Perspective Taking Test (sOPT, [34], Cronbach’s alpha = 0.80; adapted from [35]). The sOPT involves imagining standing in front of one object in a layout comprising seven objects, facing another, and pointing toward a third, indicating the direction by drawing an arrow in a circle (6 items; 5-min time limit). The score corresponds to the mean angular difference between the correct answers and the answers given (score 0–180°).

Sense of Direction–preference for survey mode scale (SOD, adapted from [34,36], Cronbach’s alpha = 0.82). This scale assesses sense of direction and preference for survey mode in the mental representation (e.g., “Do you think you have a good sense of direction?”; 6 items) rated on a Likert scale from 1 (not at all) to 5 (very much) (max 30).

Spatial anxiety scale (SA, [34], Cronbach’s alpha = 0.87; adapted from [19]). The SA assesses the degree of anxiety experienced in wayfinding situations (e.g., “Going to an appointment in an unfamiliar part of the city”; 8 items) on a Likert scale from 1 (not at all) to 6 (very much), and the sum of the ratings for all items is calculated (max 48).

#### 2.2.2. Map Learning and Recall Measures

**Figure 1:**
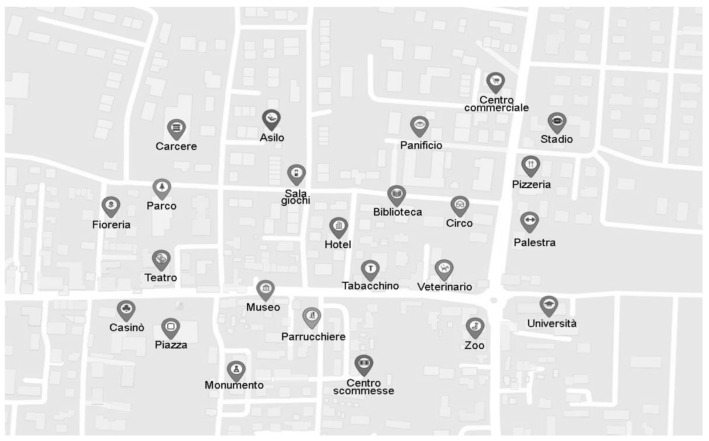
The map used in the map learning phase. The map comprises the following landmarks: bakery shop (“Panificio”), betting center (“centro scommesse”), casino (“casinò”), circus (“circo”), flower shop (“fioreria”), game room (“sala giochi”), gym (“palestra”), hairdresser (“parrucchiere”), hotel (“hotel”), kindergarten (“asilo”), library (“biblioteca”), monument (“monument”), museum (“museo”), park (“parco”), the pizzeria (“pizzeria”), prison (“prigione”), shopping center (“centro commerciale”), square (“piazza”), stadium (“stadio”), theatre (“teatro”), tobacco shop (“tabacchino”), university (“università”), veterinarian (“veterinario”), zoo (“zoo”).

Map. A map of a fictitious city was prepared in A4 format. The map contained a total of 24 landmarks depicted by their written description and a correspondent icon (see Figure 1).

Free recall of the landmarks. This involved verbally freely recalling the names of as many landmarks as possible (no time limit).

Map drawing task. This involved placing as many of the landmarks as possible in relation to one another on a white sheet of paper (A4 format) without rotating the paper. Participants were provided with a list of all the landmarks and asked to place the ones for which they felt confident about (no time limit). The number of landmarks missed, the canonical accuracy (a measure comparing each landmark position to all the other landmarks using canonical directions, NSEW, i.e., up/low and right/left with respect to other landmarks), and the inter-landmark distance accuracy (e.g., near/far correctly depicted) of each depicted landmark were retrieved from the Gardony Map Drawing analyzer (GMDA, [37]). As an example of the two latter measures, the square (see map in Figure 1) is northwest of the monument, and they have a defined distance between them. A participant who depicted the square northwest of the monument was correct in the canonical comparisons (N/S and E/W); however, the inter-landmark distance could be depicted as longer/shorter than in the target environment. The canonical accuracy scores were then summed and divided by the number of landmark comparisons. The proportional measure ranged from 0 to 1 (with increasing values meaning better positioning). The distance accuracy considered the magnitude of the inter-landmark distance error. The software calculated the distance ratio difference scores between the map drawing and the target environment (inter-landmark Euclidean distances divided by their maximum distances) and then the absolute value of each difference score. These scores were then summed and divided by the number of comparisons, and this error score was subtracted from 1. This proportional measure thus ranged from 0 to 1, with increasing values meaning a better inter-landmark distance accuracy (see [37] for more details).

### 2.3. Procedure

In the first session (lasting 40 min), participants individually completed a sociodemographic questionnaire and the MoCA, followed by the JPT, sMRT, sOPT, SOD, and SA in a balanced order. During the second session (lasting 20 min), they learned the map (five minutes) with instructions to learn the elements in the map and their locations, given that they would be then asked to solve tasks on them without the map. The map was oriented as in Figure 1; no cardinal direction was provided. The participants studied it on their own. After the learning phase, the experimenter spoke about typical conversational subjects (e.g., the weather, for about 30 s) and then asked the participants to freely recall the landmarks on the map (verbally reported without time constraints) and to perform the map drawing task (without time constraints).

## 3. Results

The data analysis was conducted with the R software [38].

### 3.1. Age-Related Differences in the Free Recall and Map Drawing Indexes

First, to analyze age-related differences in the free recall and in the canonical and distance accuracy in the map drawing task (see descriptive statistics in Table 1), linear models were run stepwise (as in [6]). Gender and education were entered into a baseline model (Step 0; given that they both related to environment learning [39,40]) to examine the group effect in relation to spatial performance after accounting for their role. Then, the age group comparing young vs. young-old and young-old vs. old-old individuals was entered in Step 1. Changes in R^2^ are reported. For all models, the variance inflation factors revealed no significant multicollinearity (VIF values ≤ 1.35).

**Figure 2:**
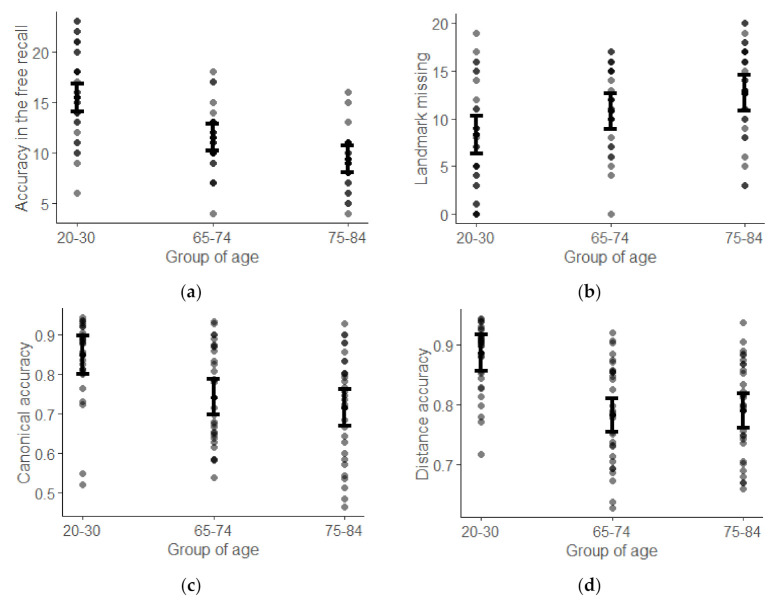
Age group effect in the regression models. (**a**) Groups of ages for free recall; (**b**) groups of ages for map drawing–missing landmark; (**c**) groups of ages for canonical accuracy; and (**d**) groups of ages for distance accuracy.

For free recall, Step 0 accounted for 22% of the variance, with education level (β = 0.44, *p* < 0.01) emerging as a significant predictor, and Step 1 accounted for another 23% of the variance, with age group emerging as a significant predictor of the free recall performance, with young adults being better than older adults (β = −0.85, *p* < 0.001) and young-old individuals being better than old-old individuals (β = −0.48, *p* = 0.017; see Figure 2a).

For the map drawing task, regarding the number of missing landmarks, Step 0 accounted for 10% of the variance, with education level emerging as a significant predictor (β = −0.31, *p* = 0.003), and Step 1 accounting for another 9% of the variance; however, age group did not emerge as a significant predictor (young vs. young-old, β = 0.46, *p* = 0.09; young-old vs. old-old, β = 0.35, *p* = 0.15; see Figure 2b). For canonical accuracy, Step 0 accounted for 2% of the variance, with no factor emerging as a significant predictor, and Step 1 accounted for 15% of the variance, with young individuals being better in positioning landmarks than older adults (β = −0.83, *p* = 0.003), but young-old and old-old individuals did not exhibit any further differences (β = −0.20, *p* = 0.40; see Figure 2c). For distance accuracy, Step 0 accounted for 4% of the variance, with no factor emerging as a significant predictor, and Step 1 accounted for 23% of the variance, with young individuals having better distance accuracy than older adults (β = −1.21, *p* < 0.001), but young-old and old-old individuals did not exhibit any further differences (β = 0.08, *p* = 0.72; see Figure 2d).

### 3.2. The Relation between Individual (Cognitive and Self-Reported) Measures and Spatial Performance

In the following analyses, we considered only the sample including older adults (*N* = 60). Correlations between age, education level, cognitive level (MoCA scores), VSWM (JPT scores), visuospatial factors (sMRT and sOPT), and self-reported wayfinding (SOD and SA) and spatial performance (free recall accuracy and map drawing accuracy—number of missing landmarks, canonical accuracy, and distance accuracy) were calculated (see Table 1). MoCA scores were correlated (|*r*| ≥ 0.37, *p* < 0.001) with free recall of landmarks, canonical accuracy, and distance accuracy in the map drawing tasks. Other weaker correlations emerged (see Table 1). With an explorative purpose, we also examined the relationship between the components of the MoCA scores with the recall task indices (free recall of landmarks, map drawing: missing landmarks, canonical accuracy, distance accuracy). The free recall of landmarks correlated with executive/visuospatial tasks (short trail-making B, cube copy, and clock-drawing tasks; r = 0.38, *p* < 0.001), naming task (r = 0.38, *p* < 0.001), serial subtraction task (r = 0.30, *p* = 0.02), and phonemic fluency task (r = 0.33, *p* = 0.01). The number of missing landmarks correlated with the digit forward-backward task (r = 0.39, *p* < 0.01). The canonical accuracy correlated with executive/visuospatial tasks (short trail-making B, cube copy, and clock-drawing tasks; r = 0.27, *p* = 0.04), the digit forward-backward task (r = 0.46, *p* < 0.01), and delayed memory recall (r = 0.41, *p* < 0.01). The distance accuracy correlated with executive/visuospatial tasks (short trail-making B, cube copy, and clock-drawing tasks; r = 0.27, *p* = 0.04), the digit forward-backward task (r = 0.43, *p* < 0.01), the repetition of two syntactically complex sentences (r = 0.29, *p* = 0.03), and delayed memory recall (r = 0.40, *p* < 0.01). In the subsequent analyses, only the total score of the MoCA was considered, however, due to a power issue.

**Figure 3:**
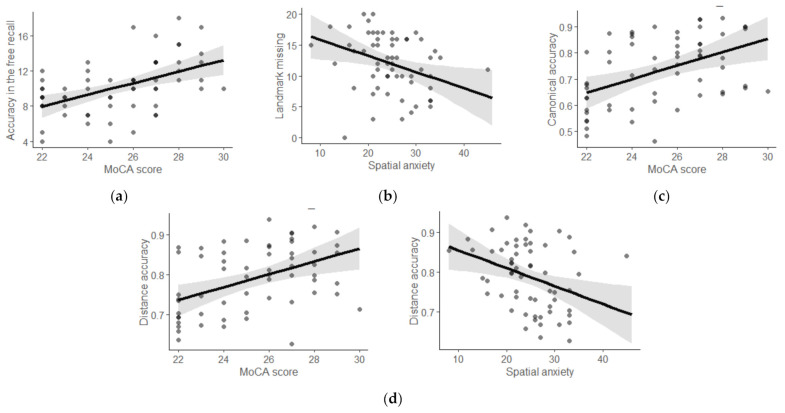
Significant predictors’ effects in the regression models. (**a**) MoCA scores for free recall; (**b**) spatial anxiety in map drawing–missing landmark; (**c**) MoCA scores for canonical accuracy; and (**d**) MoCA scores and spatial anxiety for distance accuracy.

To shed more light on the effect of the various individual factors in spatial recall (free recall, map drawing: missing landmarks, canonical accuracy, distance accuracy) in aging individuals, linear models were run stepwise to see whether the factors added into each step improved the model (changes in R^2^ are reported [26]). Gender, age, and education were entered in a baseline model (Step 0) to examine the other individual factors related to spatial performance after accounting for their role. Then, the objective factors (i.e., the MoCA as a measure of general cognitive functioning, the JPT as VSWM, and sOPT and sMRT as measuring visuospatial ability) were input in a subsequent model (Step 1). Next, self-reported wayfinding inclinations (SOD and SA) were added in a further model (Step 2) to see whether they still had a role after accounting for all of the other factors investigated (as in [26]). For all models, the variance inflation factors revealed no significant multicollinearity (VIF values ≤ 1.67). Table 2 shows the changes in R^2^, estimates, and *p*-values for all steps in all the spatial measures. For the free recall, age was a significant predictor in the baseline model, and the cognitive level (MoCA scores) resulted a predictor in the subsequent step (see Figure 3a); no factors emerged as significant predictors in Step 2.

In the map drawing task for the number of missing landmarks, no predictor emerged as significant in Steps 0 and 1; however, spatial anxiety was a predictor in Step 2 (higher level of spatial anxiety predicting lower number of missing landmarks; see Figure 3b). For the canonical accuracy, only MoCA scores were a significant predictor of the accuracy in Step 1 (see Figure 3c). For the distance accuracy, education level in Step 0, MoCA scores in Step 1, and spatial anxiety (higher level of spatial anxiety predicting lower distance accuracy) in Step 2 were significant predictors of accuracy (see Figure 3d).

## 4. Discussion

The present study aimed to investigate the characteristics of mental representation, in terms of recall about landmarks, their positions (categorical relations), and distances (distance relations) after learning a map by comparing young, young-old, and old-old participants. The quality of the representation was also investigated in relation to the role of older adults’ objective cognitive functioning (the cognitive level as measured with the MoCA test, VSWM, and rotation and perspective-taking abilities) and self-reported wayfinding inclinations (sense of direction and spatial anxiety).

Considering age-related differences after learning a new environment from a map, our results show differences between young and older adults in the number of freely recalled landmarks (i.e., recalling information about landmarks), with an additional worsening between young-old and old-old individuals. Our results also show differences in both canonical and distance accuracy, with no worsening between young-old and old-old individuals. No age-related differences were found in the number of landmarks missing in the map drawing task (recalling information about position of landmarks). These results suggest a general memory impairment with aging [29], reflecting the difficulty in recalling landmarks, with a worsening with increasing age (e.g., [30]). However, when the list of the landmarks was provided, the older adults missed as many landmarks as the young adults did in the map drawing task (after controlling for gender and education), suggesting that providing a cue mitigates the free recall difficulties [41]. Considering the positions of landmarks (above/below, left/right with respect to the other landmarks) and their distances (near/far from) in the map drawing task, the results expand the knowledge obtained using other types of knowledge acquisition [10,11] after map learning (figural scale input [2]). Considering the amount of variance explained (age effect accounted for 23% of variance in distance accuracy and 15% in canonical accuracy), our results seem to confirm a stronger age-related difference for distance relations than for categorical relations [10,11], even after the learning of a map. Underlying neuronal mechanisms may elucidate this difference. Indeed, partially distinct neuronal pathways encode categorical (relying more on the left hemisphere) and distance (relying more on the right hemisphere) spatial relations [42], as corroborated using brain imaging studies and studies of clinical populations [43,44,45,46]. Pathways could be susceptible to increasing age in different ways [47]. Therefore, future research could apply neuroscientific methods to deeply analyze categorical and distance relationships after map learning in aging. It is also important to note that we did not find a worsening between young-old and old-old individuals in both canonical and distance accuracy. This suggests that the aging issue could already be present in young-old people, and that further deterioration does not occur with increasing age. This highlights the importance of studying spatial representation characteristics even before old age [24]. Future studies are needed to better elucidate this aging issue, however.

Considering factors related to the old groups (young-old and old-old) in the various performance measures can help us to deeply understand this age-related issue in spatial mental representations derived from learning a map. Our results newly show different involvement of certain individual factors depending on the characteristics of the mental representation considered. In particular, the role of cognitive level as measured with the MoCA test emerged. Indeed, higher MoCA scores related to the number of freely recalled landmarks, the canonical accuracy (categorical relations), and the distance accuracy (distance relations). These results extend the role of cognitive functioning in aging, not only after learning an environment from navigation [25,27], but also after learning a map. The role of the general cognitive level as measured with the MoCA score emerged even when the task was not as demanding, such as map drawing after map learning. This task did not require any change in perspective [23], but rather tested allocentric knowledge [24]. This suggests that the general cognitive level as measured with the MoCA score is implied in forming a mental representation of quality per se in terms of the number of landmarks as well as their positions and distances. This was found even after learning from a map, a spatial leaning input that presents spatial information allocentrically, all at once, from one point of view. In other words, this result seems to newly show that cognitive functioning influences the mental representation of an environment in aging, regardless of the modality of information acquisition (navigation or map). This points to the importance of preserving older adults’ cognitive abilities, for instance with the aid of training programs [48], starting early in young-old adults. The other visuospatial cognitive factors considered in this study seem not to contribute to a more refined mental representation produced from map learning, although some correlations were found, even if low (e.g., VSWM correlation with free recall performance, and VSWM and perspective-taking correlation with canonical accuracy). It is worth noting, however, that it is possible that only some subcomponents of the general cognitive level as measured with the MoCA score are related to spatial performance. It is plausible that executive/visuospatial and (working memory and long-term) memory components play more prominent roles in spatial tasks. This is plausible given that map learning requires visuospatial memory processes [49], and previous studies have shown a relationship between working memory and spatial tasks [26,50]. The different involvement of the subtests of MoCA scores is an important aspect that merits being explored in future studies.

Furthermore, it is worth noting the role of education in the quality of spatial mental representation after the learning of a map. In particular, education level is related to the accuracy of freely recalling a landmark and the number of missing landmarks. This implies that education level is an important factor to consider when analyzing older adults’ spatial performance (e.g., [24]). However, this also implies the need to promote lifelong learning, even in older age [51], to counteract the decline in mental representation abilities.

On the other hand, it is worth highlighting how spatial anxiety has contributed to the quality of the mental representation after map learning. After controlling for the other individual factors, older adults’ higher level of spatial anxiety was detrimental for accurately detecting the distances between landmarks. This result expands the knowledge about spatial anxiety implied in decreasing spatial knowledge in young (e.g., [52,53]) and older adults [54], newly showing that spatial anxiety is associated with decreasing the processing of the distances between landmarks. Representing the distances between landmarks was found to be related to the emotional value of the landmarks [55]; in particular, distances between landmarks with a negative value were represented less accurately than distances between positive and neutral landmarks. Thus, it could be plausible that distance accuracy might also be related to the emotional state of the older individual (i.e., people assigning greater negative value to spatial performance being less precise in detecting distances). This interpretation merits further investigation in future studies. However, promoting positive wayfinding inclinations and lowering the level of spatial anxiety in older people could be another way to help maintain adequate spatial performance in aging.

It should be noted that, on the contrary, a certain degree of spatial anxiety led older adults to place more landmarks in the map drawing task (i.e., the higher the level of spatial anxiety, the lower the number of missing landmarks). This suggests that spatial anxiety made older adults prone to inserting more landmarks in the map drawing task, which is a non-spatial request, perhaps reflecting their general activation of attention in a task (the map drawing) that they perceive as stressful (e.g., [53,56]). However, given the limited role of visuospatial individual factors in map learning in our study, as well as the different roles played by spatial anxiety, the issue of visuospatial individual difference measures in older adults’ spatial learning merits better examination in future studies.

## 5. Conclusions

In conclusion, these results shed more light on the strengths and weaknesses of old people’s mental representation characteristics after learning a map of an environment. Knowledge of landmark information and of categorical and distance relations declines between young and older adults, with a further decline between young-old individuals and old-old individuals only in the landmark information (free recall of the landmarks). Preserving accurate knowledge about these aspects is an important prerequisite for the construction of high-quality spatial mental representations which are useful, for instance, in navigation; thus, investigating the factors related to the individual that can mitigate these difficulties is of interest. Cognitive abilities as measured with the MoCA score and personal level of spatial anxiety relate to the features of old adults’ mental representations of environments after they learn a map.

## Figures and Tables

**Table 1:** Means and standard deviation of variables in the three group of age and correlations between variables in the two older groups.

	Young M (SD)	Young-Old M (SD)	Old-Old	1.	2.	3.	4.	5.	6.	7.	8.	9.	10.	11.
1. Age	23.53 (2.36)	68.73 (3.33) *	78.50 (3.17) ^+^	-										
2. Education	13.77 (1.25)	10.73 (2.34) *	10.57 (3.46)	−0.13										
3. MoCA score	/	26.03 (2.51)	24.57 (2.05) ^+^	−0.38	−0.21									
4. Jigsaw Puzzle Test (VSWM)	22.87 (4.77)	16.20 (4.05) *	11.93 (3.34) ^+^	**−0.44**	−0.01	0.35								
5. Short Mental Rotation Test	3.70 (2.78)	1.63 (1.75) *	1.03 (1.03) ^+^	−0.18	−0.02	0.13	0.41							
6. Short Object Perspective Taking test	46.90 (37.88)	84.16 (37.67) *	103.20 (32.65) ^+^	0.23	0.09	−0.18	−0.36	**−0.44**						
7. Sense of direction and survey preference mode	15.77 (4.45)	16.67 (4.61)	15.90 (3.46)	−0.04	0.18	−0.17	−0.12	−0.03	0.28					
8. Spatial anxiety	22.93 (6.00)	24.27 (7.10)	25.03 (5.77)	−0.01	0.03	−0.13	−0.17	−0.05	−0.14	−0.41				
9. Free recall	16.03 (4.60)	11.27 (3.11) *	9.03 (2.74) ^+^	−0.38	0.25	**0.50**	0.37	0.09	−0.11	−0.01	−0.02			
10. Map drawing–Landmarks missing	7.70 (5.79)	11.07 (4.27)	13.00 (4.86)	0.16	−0.14	0.01	−0.15	0.03	0.00	−0.05	−0.26	**−0.46**		
11. Map drawing–Canonical accuracy	0.84 (0.10)	0.75 (0.12) *	0.72 (0.13)	−0.12	−0.15	**0.44**	0.17	0.13	−0.16	−0.13	−0.23	0.21	0.33	
12. Map drawing–Distance accuracy	0.88 (0.06)	0.78 (0.08) *	0.79 (0.08)	−0.01	−0.17	**0.42**	0.06	0.12	−0.08	−0.05	−0.31	0.17	0.39	**0.85**

Note. Descriptive statistics: * significant differences between young and young-old individuals, ^+^ significant differences between young-old and old-old individuals (after accounting for gender and education, except for age, education, and MoCA scores). Correlations: older groups *N* = 60; for |*r*| ≥ 0.28, *p* < 0.05; for |*r*| ≥ 0.35, *p* < 0.01, and for |*r*| ≥ 0.37, *p* < 0.001 (the latter in bold type).

**Table 2:** Regression models for free recall and map drawing index accuracy in older adults.

	Free Recall			Map Drawing—Landmark Missing			Map Drawing—Canonical Accuracy			Map Drawing—Distance Accuracy		
	ΔR^2^	β	*p*	ΔR^2^	β	*p*	ΔR^2^	β	*p*	ΔR^2^	β	*p*
Step 0 (baseline)	0.18			0.05			0.04			0.04		
Age (63–86 years old)		**−0.35**	**0.007**		0.16	0.245		−0.15	0.272		−0.01	0.964
Gender		−0.01	0.984		−0.13	0.651		0.06	0.814		−0.21	0.425
Education		0.20	0.100		−0.12	0.363		−0.17	0.211		−0.16	0.224
Step 1 (objective measures)	0.25			0.02			0.18			0.18		
MoCA score		**0.51**	**<0.001**		0.05	0.772		**0.48**	**0.002**		**0.47**	**0.002**
Jigsaw Puzzle Test (VSWM)		0.17	0.195		−0.16	0.347		−0.03	0.831		−0.09	0.81
Short Mental Rotations Test		−0.09	0.497		0.14	0.414		−0.01	0.979		0.14	0.355
Short Object Perspective Taking test		0.01	0.961		−0.01	0.943		−0.08	0.563		−0.01	0.992
Step 2 (subjective measures)	0.01			0.12			0.09			0.12		
Sense of direction and survey preference mode		−0.01	0.984		−0.20	0.225		−0.19	0.241		−0.25	0.091
Spatial anxiety		0.09	0.444		**−0.36**	**0.013**		−0.25	0.081		**−0.35**	**0.007**
R^2^	0.43			0.19			0.31			0.34		

Note. Significant standardized beta in bold type.

## Data Availability

The data presented in this study are available on request from the corresponding author.

## References

[1] Richardson A.E., Montello D.R., Hegarty M. (1999). Spatial knowledge acquisition from maps and from navigation in real and virtual environments. Mem. Cogn..

[2] Montello D.R. (1993). Scale and multiple psychologies of space. Spatial Information Theory A Theoretical Basis for GIS, Proceedings of the COSIT ’93, Elba Island, Italy, 19–22 September 1993.

[3] Iaria G., Palermo L., Committeri G., Barton J.J.S. (2009). Age differences in the formation and use of cognitive maps. Behav. Brain Res..

[4] Muffato V., Meneghetti C., Di Ruocco V., De Beni R. (2017). When young and older adults learn a map: The influence of individual visuo-spatial factors. Learn. Individ. Differ..

[5] Wilkniss S.M., Jones M.G., Korol D.L., Gold P.E., Manning C.A. (1997). Age-related differences in an ecologically based study of route learning. Psychol. Aging.

[6] Muffato V., Meneghetti C., De Beni R. (2019). Spatial mental representations: The influence of age on route learning from maps and navigation. Psychol. Res..

[7] Yamamoto N., DeGirolamo G.J. (2012). Differential effects of aging on spatial learning through exploratory navigation and map reading. Front. Aging Neurosci..

[8] Meneghetti C., Borella E., Grasso I., De Beni R. (2012). Learning a route using a map and/or description in young and older adults. J. Cogn. Psychol..

[9] Kosslyn S.M., Chabris C.F., Marsolek C.J., Koenig O. (1992). Categorical versus Coordinate Spatial Relations: Computational Analyses and Computer Simulations. J. Exp. Psychol. Hum. Percept. Perform..

[10] Bruyer R., Scailquin J.-C., Coibion P. (1997). Dissociation between Categorical and Coordinate Spatial Computations: Modulation by Cerebral Hemispheres, Task Properties, Mode of Response, and Age. Brain Cogn..

[11] Lopez A., Germani A., Tinella L., Caffò A.O., Postma A., Bosco A. (2021). The road more travelled: The differential effects of spatial experience in young and elderly participants. Int. J. Environ. Res. Public Health.

[12] Wolbers T., Hegarty M. (2010). What determines our navigational abilities?. Trends Cogn. Sci..

[13] Ruggiero G., D’Errico O., Iachini T. (2016). Development of egocentric and allocentric spatial representations from childhood to elderly age. Psychol. Res..

[14] Kosslyn S.M. (1996). Image and Brain.

[15] Hegarty M., Montello D.R., Richardson A.E., Ishikawa T., Lovelace K. (2006). Spatial abilities at different scales: Individual differences in aptitude-test performance and spatial-layout learning. Intelligence.

[16] Logie R.H. (1995). Visuo-Spatial Working Memory.

[17] Vandenberg S.G., Kuse A.R. (1978). Mental rotations, a group test of three-dimensional spatial visualization. Percept. Mot. Ski..

[18] Hegarty M., Waller D. (2004). A dissociation between mental rotation and perspective-taking spatial abilities. Intelligence.

[19] Lawton C.A. (1994). Gender differences in way-finding strategies: Relationship to spatial ability and spatial anxiety. Sex Roles.

[20] Borella E., Meneghetti C., Ronconi L., De Beni R. (2014). Spatial abilities across the adult life span. Dev. Psychol..

[21] Kraemer D.J.M., Schinazi V.R., Cawkwell P.B., Tekriwal A., Epstein R.A., Thompson-Schill S.L. (2017). Verbalizing, visualizing, and navigating: The effect of strategies on encoding a large-scale virtual environment. J. Exp. Psychol. Learn. Mem. Cogn..

[22] Nasreddine Z.S., Phillips N.A., Bèdirian V., Charbonneau S., Whitehead V., Collin I., Cummings J.L., Chertkow H. (2005). The Montreal Cognitive Assessment, MoCA: A Brief Screening Tool for Mild Cognitive Impairment. J. Am. Geriatr. Soc..

[23] Bosco A., Spano G., Caffò A.O., Lopez A., Grattagliano I., Saracino G., Pinto K., Hoogeveen F., Lancioni G.E. (2017). Italians do it worse. Montreal Cognitive Assessment (MoCA) optimal cut-off scores for people with probable Alzheimer’s disease and with probable cognitive impairment. Aging Clin. Exp. Res..

[24] van der Ham I.J.M., Claessen M.H.G. (2020). How age relates to spatial navigation performance: Functional and methodological considerations. Aging Res. Rev..

[25] O’Malley M., Innes A., Wiener J.M. (2018). How do we get there? Effects of cognitive aging on route memory. Mem. Cogn..

[26] Richmond L.L., Sargent J.Q., Flores S., Zacks J.M. (2018). Age differences in spatial memory for mediated environments. Psychol. Aging.

[27] Muffato V., De Beni R. (2020). Path Learning From Navigation in Aging: The Role of Cognitive Functioning and Wayfinding Inclinations. Front. Hum. Neurosci..

[28] Lopez A., Postma A., Bosco A. (2020). Categorical & coordinate spatial information: Can they be disentangled in sketch maps?. J. Environ. Psychol..

[29] Craik F.I., Salthouse T.A. (2011). The Handbook of Aging and Cognition.

[30] Gazova I., Laczó J., Rubinova E., Mokrisova I., Hyncicova E., Andel R., Vyhnalek M., Sheardova K., Coulson E.J., Hort J. (2013). Spatial navigation in young versus older adults. Front. Aging Neurosci..

[31] Crook T., Bartus R.T., Ferris S.H., Whitehouse P., Cohen G.D., Gershon S. (1986). Age-associated memory impairment: Proposed diagnostic criteria and measures of clinical change—Report of a national institute of mental health work group. Dev. Neuropsychol..

[32] World Medical Association (2013). World Medical Association Declaration of Helsinki. JAMA.

[33] De Beni R., Borella E., Carretti B., Marigo C., Nava L.A. (2008). Portfolio per la Valutazione del Benessere e delle Abilità Cognitive nell’età Adulta e Avanzata [The Assesment of Well-Being and Cognitive Abilities in Adulthood and Aging].

[34] De Beni R., Meneghetti C., Fiore F., Gava L., Borella E. (2014). Batteria Visuo-Spaziale. Strumenti per la Valutazione delle Abilità Visuo-Spaziali nell’arco di Vita Adulta [Visuo-Spatial Battery: Instrument for Assessing Visuo-Spatial Abilities across Adult Life Span].

[35] Kozhevnikov M., Hegarty M. (2001). A dissociation between object manipulation spatial ability and spatial orientation ability. Mem. Cogn..

[36] Pazzaglia F., Meneghetti C. (2017). Acquiring Spatial Knowledge from Different Sources and Perspectives. Representations in Mind and World.

[37] Gardony A.L., Taylor H.A., Brunyé T.T. (2016). Gardony Map Drawing Analyzer: Software for quantitative analysis of sketch maps. Behav. Res. Methods.

[38] R Core Team (2020). R: A Language and Environment for Statistical Computing.

[39] Coluccia E., Louse G. (2004). Gender differences in spatial orientation: A review. J. Environ. Psychol..

[40] Ardila A., Ostrosky-Solis F., Rosselli M., Gómez C. (2000). Age-related cognitive decline during normal aging: The complex effect of education. Arch. Clin. Neuropsychol..

[41] Naveh-Benjamin M., Craik F.I.M., Ben-Shaul L. (2002). Age-Related Differences in Cued Recall: Effects of Support at Encoding and Retrieval. Aging Neuropsychol. Cogn..

[42] van der Ham I.J.M., Raemaekers M., van Wezel R.J.A., Oleksiak A., Postma A. (2009). Categorical and coordinate spatial relations in working memory: An fMRI study. Brain Res..

[43] Hartley T., Lever C., Burgess N., O’Keefe J. (2014). Space in the brain: How the hippocampal formation supports spatial cognition. Philos. Trans. R. Soc. B Biol. Sci..

[44] Postma A., van der Ham I.J.M. (2016). The Neuropsychology of Space.

[45] Ruzich E., Crespo-García M., Dalal S.S., Schneiderman J.F. (2019). Characterizing hippocampal dynamics with MEG: A systematic review and evidence-based guidelines. Hum. Brain Mapp..

[46] Rungratsameetaweemana N., Squire L.R. (2018). Preserved capacity for scene construction and shifts in perspective after hippocampal lesions. Learn. Mem..

[47] Cabeza R., Nyberg L., Park D.C. (2016). Cognitive Neuroscience of Aging: Linking Cognitive and Cerebral Aging.

[48] Lövdén M., Schaefer S., Noack H., Bodammer N.C., Kühn S., Heinze H.-J., Düzel E., Bäckman L., Lindenberger U. (2012). Spatial navigation training protects the hippocampus against age-related changes during early and late adulthood. Neurobiol. Aging.

[49] Coluccia E. (2008). Learning from maps: The role of visuo-spatial working memory. Appl. Cogn. Psychol. Off. J. Soc. Appl. Res. Mem. Cogn..

[50] Weisberg S.M., Newcombe N.S. (2016). How do (some) people make a cognitive map? Routes, places, and working memory. J. Exp. Psychol. Learn. Mem. Cogn..

[51] Merriam S.B., Kee Y. (2014). Promoting community wellbeing: The case for lifelong learning for older adults. Adult Educ. Q..

[52] Hund A.M., Minarik J.L. (2006). Getting from here to there: Spatial anxiety, wayfinding strategies, direction type, and wayfinding efficiency. Spat. Cogn. Comput..

[53] Viaud-Delmon I., Berthoz A., Jouvent R. (2002). Multisensory integration for spatial orientation in trait anxiety subjects: Absence of visual dependence. Eur. Psychiatry.

[54] Meneghetti C., Borella E., Pastore M., De Beni R. (2014). The role of spatial abilities and self-assessments in cardinal point orientation across the lifespan. Learn. Individ. Differ..

[55] Ruotolo F., Claessen M.H.G., van der Ham I.J.M. (2019). Putting emotions in routes: The influence of emotionally laden landmarks on spatial memory. Psychol. Res..

[56] Ouimet A.J., Gawronski B., Dozois D.J.A. (2009). Cognitive vulnerability to anxiety: A review and an integrative model. Clin. Psychol. Rev..

